# P53 prevent tumor invasion and metastasis by down-regulating IDO in lung cancer

**DOI:** 10.18632/oncotarget.17408

**Published:** 2017-04-25

**Authors:** Dongfang Tang, Lu Yue, Ruyong Yao, Lin Zhou, Yuqin Yang, Liming Lu, Wen Gao

**Affiliations:** ^1^ Department of Thoracic Surgery, Huadong Hospital Affiliated to FuDan University, Huadong, China; ^2^ Department of Oncology of the Qingdao Municipal Hospital, Qingdao, China; ^3^ Central Laboratory of the Affiliated Hospital of Medical College Qingdao University, Qingdao, China; ^4^ Central Laboratory of Shanghai Chest Hospital Affiliated to Shanghai Jiaotong University, China

**Keywords:** lung cancer, IDO signaling, p53, metastasis

## Abstract

In present study, we are to clear demonstrate the genetic evidence of IDO signaling's impact on invasion and metastasis in lung cancer. Here we examined IDO1 expression levels in non-small cell lung cancer (NSCLC) patients (64) tumor/normal pairs underwent RT-PCR and comprehensive histological, immunohistochemica and clinical analysis. The NSCLC cells stably expressing IDO1 was analyzed for migration and invasion assays and the regulatory mechanism *in vitro* and metastasis assays *in vivo*. As results, we reported that IDO1 expression increased by more than 3.2-fold in lung cancer compared with their corresponding non-tumor tissues, and the up-regulation of IDO1 is significantly correlated to TNM stage and lymph node-metastasis. The over-expression of IDO1 significantly encouraged the metastasis and invasion of lung cancer cells, and IDO1 could promote metastasis formation *in vivo*. Furthermore, we further found that p53 could attenuate IDO signaling in lung cancer cell migration partly. In conclusion, these results demonstrate that the IDO signaling's impact on invasion and metastasis and the suppressive effect of p53 on IDO1 in lung cancer, present one novel therapeutic strategy for early metastatic lung cancer in clinical.

## INTRODUCTION

Distant metastasis and invasion are in charge of most cancer related deaths [1]. The course of tumor metastasis compose of consequent steps including cancer cells invade into surrounding tissue, exist in circulation, stay at distant organs, and grow into tumor in the distant sites [2].

Indoleamine-2, 3-dioxygenase is a 42-45KD enzyme which catalyzes the rate limiting steps of tryptophan (Trp) degradation along the kynurenine pathway [3]. IDO-1 exerts a potent immunosuppressive effect through inhibiting T-lymphocytes and other immune cells. Additionally, IDO-1 has been shown to induce immunosuppressive effects and favor tumor progression in animal models of lung cancer [4, 5]. Variable levels of IDO-1 have been found in human solid tumors including melanomas, gliomas, and carcinomas from different locations. Blockade of IDO-1 using small molecule inhibitors in combination with immune checkpoint blockade induces prominent antitumor responses in mouse models and reversal of tumor-associated immunosuppression by 1methyl-D-tryptophan appears to be dependent on host IDO1 expression [6]. Whereas, evident proof for IDO1's influence on invasion or metastasis in lung cancer is lacking.

P53 suppresses the expression of numerous target genes as a master regulatory suppressor gene directly or indirectly, which block cell proliferation or activate cell death programs in suppression of tumor development and growth [7]. Currently, we found that IDO1 expression increased in lung cancer contrast with the corresponding non-tumor tissues, and the up-regulation of IDO1 is associated with TNM stage and lymph node-metastasis. In addition, we further found that IDO pathway is involved in NSCLC cell migration and invasion and p53 could negatively modulate IDO signaling partly. These results present a therapeutic strategy for early metastatic lung cancer.

## RESULTS

### IDO1 is associated with advanced clinical stage and lymph node-metastasis

**Figure 1:**
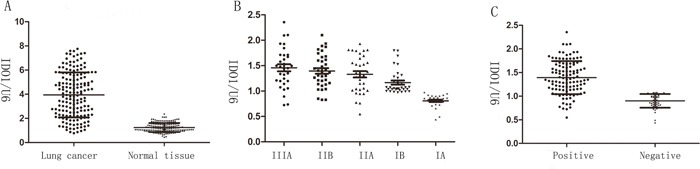
IDO1 is up-regulated in lung cancer and associated with advanced clinical stage and lymph node-metastasis **(A)** IDO1 expression of was determined by way of qRT-PCR in 64 paired human lung cancer and their corresponding nontumorous samples (NT) and normalized against an endogenous U6 RNA control. **(B)** IDO1expression in different clinical stages of lung cancer. Patients were staged in accordance with the 8th Edition of the TNM staging for lung cancer of UICC. **(C)** up-regulation of IDO1 in lung cancer was associated with lymph node-metastasis; patients were classified into lymph node-metastasis negative group (LN-negative) and positive group (LN-positive).

Firstly, we determined the IDO1 expression levels in 64 pairs of primary lung cancer and their corresponding non-tumorous tissues by qRT-PCR. Table 1 showed the relationship between the IDO1 expression levels and clinicopathologic characteristics. The results revealed that there were no significant relationship between the IDO1 expression and age, tumor size, location, gender, local invasion, and differentiation. Whereas, we discovered that IDO1 expression was up-regulated by more than 3.2-fold in lung cancer compared with non-tumorous tissues (Figure 1A). We also found that advanced stages (stage III) had a higher IDO1 expression than early stages in lung cancer (stage I and II; Table 1 and Figure 1B). Interestingly, when 64 lung cancer samples were stratified according to the lymph node-metastasis status (Postoperative lymph node examination was positive), we found that IDO1 was significantly up-regulated in lung cancer that had lymph node-metastasis (P <0.001; Figure 1C). Collectively, the above discoveries suggest that IDO1 expression may exert a significant function in lung cancer metastasis.

**Table 1:** The relationship between IDO1 expression and clinicopathological characteristic in Lung cancer patients

Variate	Number	Median expression level	P
Age			
≤60	28	1.265±0.40	
>60	36	1.222±0.35	0.236
Gender			
Male	37	1.262±0.35	
Female	27	1.223±0.41	0.254
PS			
≤2	56	1.240±0.37	
>2	8	1.290±0.44	0.286
Smoking			
Never	33	1.250±0.38	
Ever	31	1.242±0.36	0.44
Histology			
Adenocarcinoma	29	1.220±0.36	
Squamous	27	1.309±0.43	
Other	8	1.214±0.26	0.362
Lymph-node metastasis			
N	45	0.902±0.146	
Y	19	1.393±0.35	<0.0001
Stage			
IA	16	0.806±0.12	
IB	10	1.166±0.24	
IIA	13	1.330±0.36	
IIB	17	1.396±0.33	
IIIA	8	1.458±0.39	<0.001*

Note:* represents both group 1(I) VS group 2 (II, III) and group 3(I, II) VS group 4 (III).

### IDO1 promotes cell invasion and metastasis *in vitro* and *vivo*

**Figure 2:**
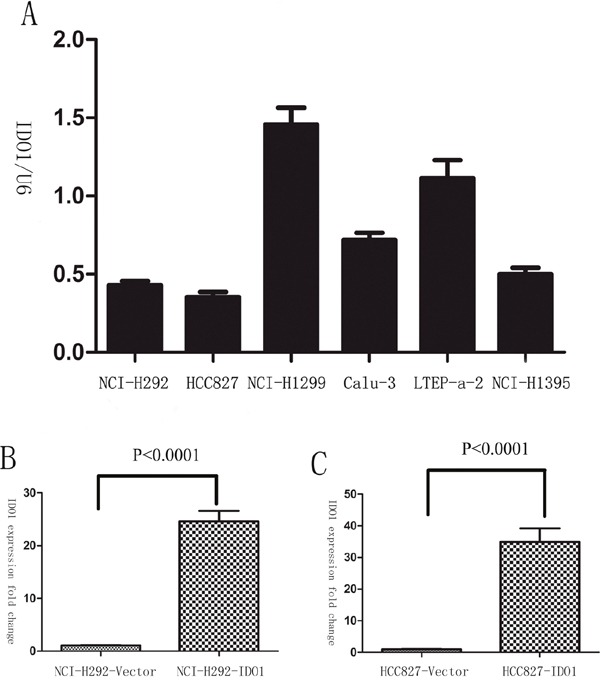
**(A)** The expression levels of IDO1 were determined by qRT-PCR in lung cancer cell lines. U6 RNA served as an internal control. **(B)** And **(C)** Successful over-expression of IDO1 was confirmed by qRT-PCR after infection with IDO1-expressing lentivirus or vector control lentivirus. The values of IDO1 expression were calculated as fold change relative to the vector control.

**Figure 3:**
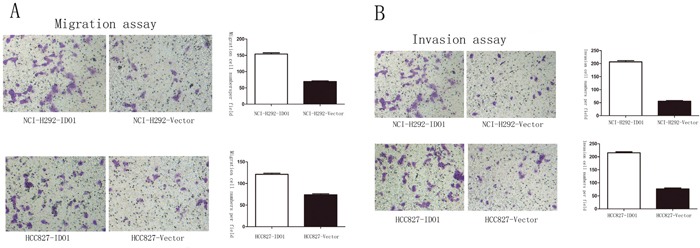
Overexpression of IDO1 significantly enhanced abilities of cell migration **(A)** and invasion **(B)** in NCI-H292 and HCC827 Lung cancer cell after infection with IDO1-expressing or vector lentivirus.

**Figure 4:**
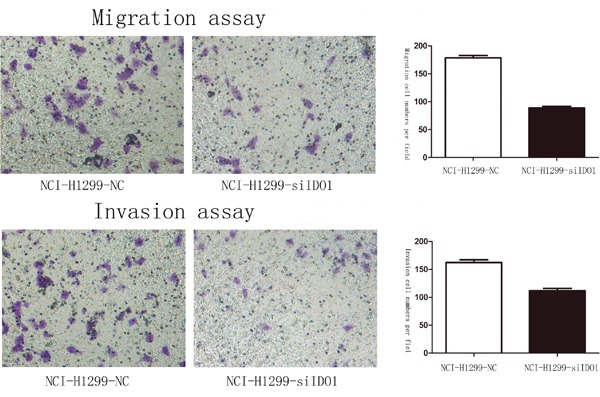
Downregulation of IDO1 significantly suppressed cell migration and invasion in NCI-H1299 Lung cancer cell

**Figure 5:**
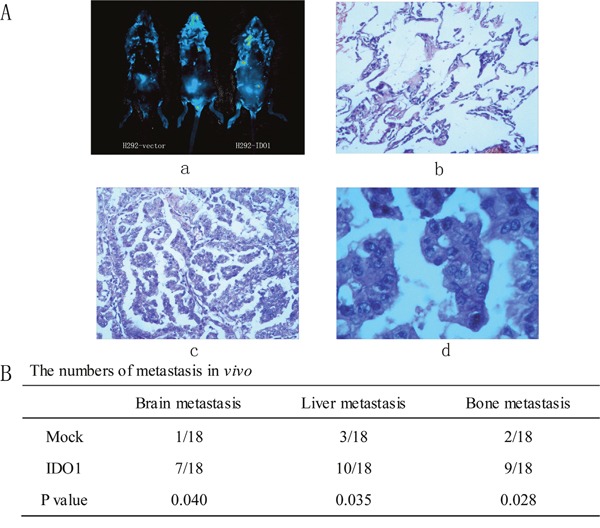
Representative H&E-stained sections of the lung tissues isolated from mice that seeded into lungs with NCI-H292-IDO1 or NCI-H292-Vector cells directly **(a)** Successfully transplanted nude mouse, migration to peritoneal cavity and control nude mouse. **(b)** Normal lung tissue. **(c)** and **(d)** cancer tissue for 10 ×10 and 40×10 horizon. **(B)** The numbers of metastasis *in vivo*.

Because we observed that the up-regulation of IDO1 in lung cancer is a frequent event and closely associated with lung cancer metastasis, we postulated that increased IDO1 in lung cancer cells may impose positive effects on cell invasion and metastasis. Therefore, we established NCI-H292 and HCC827 cell lines that stably express IDO1 by lentivirus infection, as NCI-H292 and HCC827 cells have low basal levels of IDO1 in lung cancer cell lines (Figure 2A). Overexpression of IDO1 was verified by qRT-PCR (Figure 2B and 2C). Interestingly, we discovered that over-expression of IDO1 significantly promoted metastasis and invasion of lung cancer cells (Figure 3A and 3B). Conversely, we transfected si-IDO1 into NCI-H1299 cells, which have relatively high endogenous IDO1 expression among lung cancer cell lines (Figure 2A). We detected that knockdown of IDO1 could inhibit lung cancer cell migration and invasion (Figure 4). To further probe the role of IDO1 on tumor metastasis *in vivo*, NCI-H292 cells stably expressing IDO1 were transplanted into lungs of nude mice directly. Histological analysis confirmed that IDO1 could promote metastasis formation. The numbers and size of metastasis nodules were significantly increased in NCI-H292-IDO1 group when compared with NCI-H292-vector group (Figure 5A and Figure 5B). Taken together, our results suggest that IDO1 is a positive regulator for lung cancer metastasis.

### Up-regulation of IDO is correlated with p53 expression inversely in lung cancer

**Figure 6:**
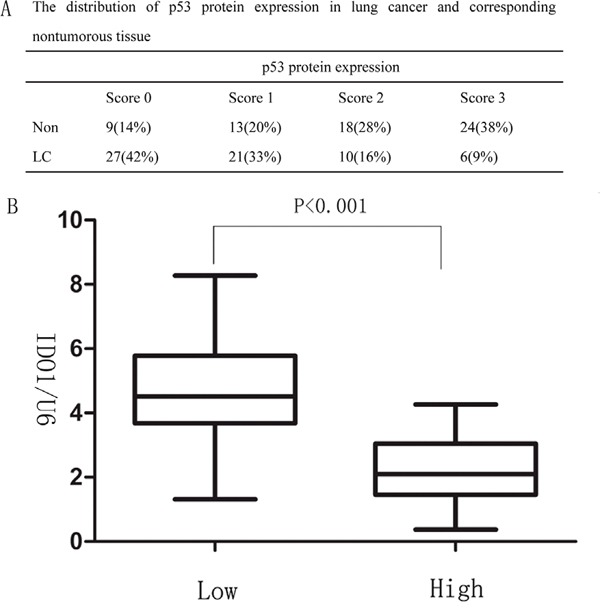
**(A)** The distribution of p53 protein expression in lung cancer and corresponding nontumorous tissue **(B)** Correlation between p53 expression and IDO1 levels in the 64 lung cancer tissue samples. The expression levels of p53 were classified into low (scores of 0 and 1) and high groups (scores of 2 and 3) according to the scores of p53 immunohistochemical staining.

As p53 is well-known suppressor gene and down-regulated in lung cancer, we next determined whether IDO1 is negatively correlated with p53 levels in the lung cancer tissue samples. Analysis of p53 expression in lung cancer and their corresponding non-tumorous tissues by immunohistochemisty staining showed that p53 proteins were significantly down-regulated in lung cancer (P <0.001; Figure 6A). Furthermore, we found that high expression of IDO1 proteins were more likely to be seen in lung cancer with low level of P53 (P < 0.001; Figure 6B), suggesting that the up-regulation of IDO1 proteins may result from repression of p53 in lung cancer.

### p53 suppress the role of IDO signaling in cell migration and invasion

**Figure 7:**
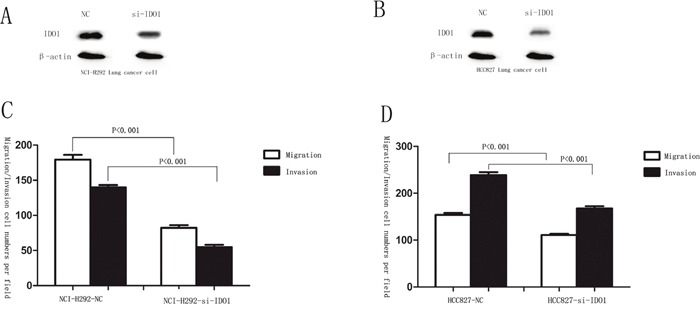
p53 attenuates IDO1 in lung cancer cell migration and invasion **(A)** and **(B)** silencing of IDO 1 was confirmed by Western blot in NCI-H292 and HCC827 cells after transfection with specific si-IDO1. β-Actin served as an internal control. **(C)** and **(D)** migration and invasion assays were carried out in NCI-H292 and HCC827 cells after transfection with negative control (NC) or si-IDO1.

**Figure 8:**
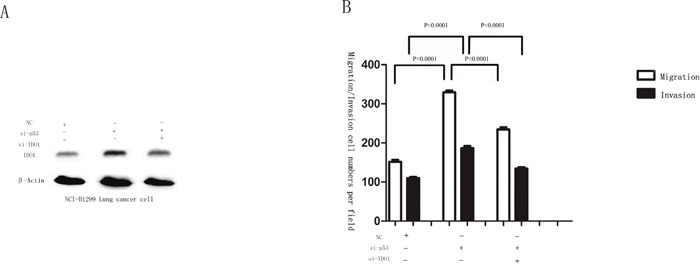
**(A)** Western blot analysis was used to detect the IDO1 expression in NCI-H1299 cells after transfection with si-p53, si-IDO1 or NC. β-Actin served as an internal control. **(B)** NCI-H1299 cells after transfection with si-p53, si-IDO1 or NC were subjected to migration and invasion assays.

Migration and invasion assays showed that si-IDO1 could counteract lung cancer cell migration and invasion (Figure 7A, 7C and Figure 7B, 7D), which is similar with the inhibitory effects of p53 on the lung cancer cell migration and invasion. Just like the observation that p53 is down-regulated in lung cancer; p53 silencing enhances IDO1 expression in lung cancer cells. To determine whether deregulation of IDO1 is involved in regulation of cell migration and invasion by p53, we cotransfected NCI-H1299 with si-p53 and si-IDO1. (Figure 8A) As a result, we found that the invasion-promoting effects of anti-p53 were partially attenuated by si-IDO1 (Figure 8B), suggesting that IDO signaling is one of the modulation pathways inhibited by p53 and p53 could prevent cell invasion and migration by down-regulating IDO1.

## DISCUSSION

Lymph node-metastasis is an initial step of lung cancer metastasis, and is essential in the determination of the clinical stage, prognosis, and survival. Therefore, identifying metastatic factors and elucidating the molecular mechanisms underlying lymph node-metastasis become critical [8]. Immune escape represents a groundbreaking though most untested paradigm in the field of cancer biology [9–11]. Initially, Munn et al. assumed that cancers exploit IDO activity as a mechanism of immune escape on maternal immune tolerance [12]. Despite of fundamental deficit in genetic support, it has become accepted increasingly of IDO's role in tumor development. In present study, our discovery revealed that high IDO1 expression was significantly correlated with lymph node-metastasis, as the lung cancer with lymph node-metastasis had a much higher IDO1 expression than those without lymph node-metastasis. In addition, our functional studies further found that IDO1 played a major role in lung cancer metastasis, and overexpression of IDO1 could promote migration and invasion *in vitro* and metastasis *in vivo*. Taken together, these data indicates that IDO1 has a close relationship with lymph node-metastasis and acts as metastatic modulator in lung cancer.

Tumor cells move either as individual cells or via collective migrationin the cancer invasion–metastasis cascade [12]. Alexander J. Muller et al. have found that a function of IDO signaling in the development of vascular under native physiological conditions [9]. And Jie Mei et al. have reported that IDO1-overexpression in endometrial stromal cell was markedly linked to up-regulation of invasion, proliferation, adhesion, as well as expression of MMP-9, COX-2 expression [13]. Likewisely, Chang Su et al. have found that Erianin may inhibit angiogenesis induced by IDO through the inhibition of JAK2/STAT3 phosphorylation and the expression of its downstream target genes MMP-2 and MMP-9. At the same time, overexpression of IDO can increase the expression of inflammatory medium in 2LL cells and the promoting effect of COX-2 is particularly significant [14]. It appears that IDO1 pathway plays an essential function in cancer invasion and metastasis by promoting angiogenesis and inflammatory response. Our findings demonstrated the important role of IDO activity in sustaining lung metastasis genetically. IDO activity may influence metastatic dissemination to tissues like the lung. Whereas, it is less relevant when IDO activity is most increased within the tumor cells, enabling the malignancy to shape its microenvironment through intrinsic tryptophan catabolism [15]. However, where is IDO1 exactly coming from? Karanikas et al. found [16] that the expression of IDO was significantly lower in lung cancer cell lines than that of patients' tumor samples and the autologous non-affected lung tissues. Therefore, they considered that the IDO owns to the production of the enzyme by other cells recruited in the tumor microenvironment. After all, if IDO1 is derived from the tumor, the expression of IDO1 in cultured cells cannot be lower than that of the autologous non-affected lung tissues. However, we do not object their opinions, we believe that IDO1 may derive from normal cells, or it is possible that IDO1 is one of the tumor's autocrine hormones. As such, IDO activity that originates from the tumor cells themselves or from stromal cells of the tumor microenvironment may contribute to directing tumor outgrowth.

The present study examined whether p53 protein affects the expression level of IDO signaling to further investigate the underlying mechanisms. Mei J et al. also have reported [13] that IDO1 regulated p53 expression in normal ESCs via JNK signaling pathway. In our study, we found that the up-regulation of IDO1 proteins may result from repression of p53 in lung cancer based on large and enriched experimental data. In order to demonstrate their results, they carried out the experiments in three steps. Firstly, they compared vector control without SP600125 with IDO1-overexpressing ESCs without SP600125 (JNK pathway inhibitor), they found that the expression of p53 was suppressed to 77.1%, we concluded that IDO1 and P53 expression were inversely based on this step; Secondly, they compared IDO1-overexpressing ESCs with SP600125 with IDO1-overexpressing ESCs without SP600125, they found that the expression of P53 was elevated to 117%; we concluded that JNK pathway has an inhibitory effect on P53 based on this step. In the third part, they compared IDO1-deficency ESCs with and without SP600125; they found that p53 expressions were stimulated to 185% and 190%. However, if the above two steps’ results have no problem, in this part, their achieved results were contradictory. For if IDO1 regulated p53 expression via JNK signaling pathway, the p53 expression in IDO1-deficency ESCs with SP600125 will be higher obviously than that without SP600125, while they found that the p53 expression in IDO1-deficency ESCs with SP600125 was lower. In our study, we found that the up-regulation of IDO1 proteins may result from repression of p53 in lung cancer base on large and enriched experimental data. Therefore, we confirm that IDO1 pathway is one of the downstream targets of P53. In conclusion, we have revealed a novel pathway mechanism suggesting the following suggestion: loss the capacity of p53 activates IDO1 signaling transcription which promotes lung cancer cell invasion and metastasis. It may provide novel therapeutic targets against lung cancer progression.

## MATERIALS AND METHODS

### Human samples

Human lung cancer and their corresponding non-tumorous tissues were collected instantly after surgery from 64 patients with NSCLC from April 2015 to October 2015 at the Department of Thoracic Surgery of the HuaDong Hospital Affiliated to FuDan University. Human tissues were frozen in liquid nitrogen and stored at -80°C refrigerator promptly. All patients signed informed consent and the study was approved by the Clinical Research Ethics Committee of FuDan University.

### Cell culture

Human non-small-cell lung cancer cell lines (NCI-H292: pulmonary carcinoma cell line, HCC827: human lung adenocarcinoma cell line, NCI-H1299: non-small cell lung cancer cell line), and HEK293T cells were purchased from the Cell Resource Center, Shanghai Institute of Biochemistry and Cell Biology at the Chinese Academy of Sciences (No.220 Yueyang Road, Xuhui District, Shanghai, China). Cells were cultured in a humidified air atmosphere at 37°C, RPMI1640 (NCI-H292, HCC827), F12 (NCI-H1299) containing 5% carbon dioxide [17].

### Quantitative RT-PCR

Total RNA was extracted using the Recover All Total Nucleic Acid Isolation Kit (Ambion; Life Technologies) [18]. cDNA was synthesized from total RNA with specific stem-loop primers and the TaqMan Reverse Transcription Kit (Applied Biosystems; Life Technologies).

The primers sequences were as follows: IDO1, forward, 5'-GGGGACAAGTTTGTACAAAAAAGCAGG CTGCCACCATGGCACTCAGTAAAATATCTCCT -3′; Reverse, 5′- GGGGGAGGGAGAGGGGCCTAAGGCCA ACTCAGAAGAGC -3′; p53, forward: 5'-CCAGGATGT TGCAGAGTTGTTA-3', Reverse: 5'-CTCACGACCTCA GTCATGTGTT-3'; Using β-Actin as an internal control, forward primer: 5′-AGTGTGACGTGGACA TCCGCAAAG-3′, and reverse primer: 5′-ATCCACATCTGCTGGAAGGTGGAC-3′.

### Vector constructs

Both pri- and 3’ UTR sequences of IDO1, p53 were amplified from normal human genomic DNA, the pri-IDO1, p53 sequence was constructed into the lentivirus expression vector pWPXL to generate pWPXL- IDO1, p53 [19]. The 3’UTR sequence was subcloned into the region directly downstream of a cytomegalovirus (CMV) promoter driven firefly luciferase cassette in a pCDNA3.0 vector (pLuc). With appropriate primers, a series of 3’ UTR sequences were generated and inserted into the pCDNA3.0 vector.

### Oligonucleotide transfection

siRNA against IDO1 were synthesized by Ribobio. The sequence of siRNA targeting IDO1 is: GAACCCACTGCTTACTGGCTT and p53 as follows GCAUGAACCGCCGGCCCAUTT. Lipofectamine™ 2000 (Invitrogen) reagents was used in Oligonucleotide transfection.

### Lentivirus transduction

pWPXL-IDO1, pWPXL-p53 or pWPXL were transfected into NCI-H292 cells using lipofectamin 2000 reagent to generate lentivirus. HCC827 and NCI-H1299 cells were infected with the recombinant lentivirus transducing units plus 6 mg/mL Polybrene (Sigma) [19].

### Migration and invasion assay *in vitro*

In transwell assays, 2.5×10^4^ to 5×10^4^ cells were plated in the top chamber with the non-coated membrane, in invasion assay, 1.25×10^5^cells were plated in the top chamber with Matrigel-coated membrane. The cells that did not shift through the pores after incubating for 24 h were removed. Cells were stained with the Crystal Violet on the lower surface of the membrane (GuangZhou Xilong Chemical industry co.) and counted.

### Animal

Six-week-old NOD-SCID mice were bought from BeiJing d tong lihua experimental animal technical co., LTD. Mice were anaesthetized with 2, 2, 2-tribromoethanol. NCI-H292 cells transfected with either the IDO1-overexpressing lentivirus or the mock lentivirus were implanted into lungs directly (1×10^6^ cells for each mouse) [20]. Each group consisted of 18 mice: 3–4 mice per group were euthanized at week 6, 9 and 11 post transplantationaccepting NCI-H292 cells with mock lentivirus; mice were euthanized when the tumors were 2 cm enough for accepting NCI-H292 cells with IDO1-overexpressing lentivirus, and the metastases were evaluated carrying primary tumors at the same point. Thus, the tumors were removed and weighed. Microscope equipped with bright field and luciferase imaging were used to andlyze tumors, ossatures, brains, livers and macroscopic metastases.

### Western blot analysis

Proteins were separated by 8% SDS-PAGE and transferred to nitrocellulose membrane (Bio-Rad). After blocking in 5% nonfat milk, the membranes were incubated with the following primary antibodies: rabbit anti-IDO1 monoclonal antibody (mAb; 1:100; Abnova), mouse anti-β-actin mAb (1:10,000; Sigma) [21]. The proteins were visualized with enhanced chemiluminescencere agents.

### Immunohistochemical staining

Immunohistochemical staining was done according to standard procedures. For incubation with primary mAb, Tissue slides were incubated at 4°C overnight with rabbit anti-IDO1 mAb (1:50; Abnova). Scoring was measured by the cell cytoplasm staining of tumor or non-tumorous tissues as described: scored 0, absent cell cytoplasm staining; scored 1, weak staining; scored 2, moderate staining; scored 3, strong staining [22]. The mice surgical samples were fixed in10% buffered formalin for 12 h, followed by a wash with PBS and transfer to 70% ethanol, embedded in paraffin, sectioned, stained with haematoxylin and eosin. Detected using rabbit anti-p53 (1:1000, GentTex) with a BioGenex i6000 automated stainer in the Department of Pathology. All research was approved by the FuDan University Experimental Animal Care Commission.

### Statistical analysis

Data were shown as mean±SD, Student t test was used for statistical analysis. SAS 9.4 software was carried out for all statistical analyses. Graph Pad Software (Graph Pad Software, Inc, La Jolla, CA) was used to show the distributions of expression [23], P<0.05 were considered to be significant.

## Abbreviations

NSCLC: non-small cell lung cancer
IDO-1: Indoleamine-pyrrole 2, 3-dioxygenase
TNM stage: Tumor node metastasis stage
ESCs: Endometrial stromal cells

## References

[1] Shimada Y, Ishii G, Hishida T, Yoshida J, Nishimura M, Nagai K (2010). Extratumoral vascular invasion is a significant prognostic indicator and a predicting factor of distant metastasis in non-small cell lung cancer. J Thorac Oncol.

[2] Zijlstra A, Lewis J, Degryse B, Stuhlmann H, Quigley JP (2008). The inhibition of tumor cell intravasation and subsequent metastasis via regulation of *in vivo* tumor cell motility by the tetraspanin CD151. Cancer Cell.

[3] Yuen HF, Chan YK, Grills C, McCrudden CM, Gunasekharan V, Shi Z, Wong AS, Lappin TR, Chan KW, Fennell DA, Khoo US, Johnston PG, El-Tanani M (2011). Polyomavirus enhancer activator 3 protein promotes breast cancer metastatic progression through Snail-induced epithelial-mesenchymal transition. J Pathol.

[4] Isla Larrain MT, Rabassa ME, Lacunza E, Barbera A, Cretón A, Segal-Eiras A, Croce MV (2014). IDO is highly expressed in breast cancer and breast cancer-derived circulating microvesicles and associated to aggressive types of tumors by in silico analysis. Tumour Biol.

[5] Schalper KA, Carvajal-Hausdorf D, McLaughlin J, Altan M, Velcheti V, Gaule P, Sanmamed MF, Chen L, Herbst RS, Rimm DL (2016). Differential Expression and Significance of PD-L1, IDO-1, and B7-H4 in Human Lung Cancer. Clin Cancer Res.

[6] Opitz CA, Litzenburger UM, Opitz U, Sahm F, Ochs K, Lutz C, Wick W, Platten M (2011). The indoleamine-2,3-dioxygenase (IDO) inhibitor 1-methyl-D-tryptophan upregulates IDO1 in human cancer cells. PLoS One.

[7] Zhang P, Tu B, Wang H, Cao Z, Tang M, Zhang C, Gu B, Li Z, Wang L, Yang Y, Zhao Y, Wang H, Luo J (2014). Tumor suppressor p53 cooperates with SIRT6 to regulate gluconeogenesis by promoting FoxO1 nuclear exclusion. Proc Natl Acad Sci USA.

[8] Koike T, Koike T, Yamato Y, Yoshiya K, Toyabe S (2012). Predictive risk factors for mediastinal lymph node metastasis in clinical stage IA non-small-cell lung cancer patients. J Thorac Oncol.

[9] Smith C, Chang MY, Parker KH, Beury DW, DuHadaway JB, Flick HE, Boulden J, Sutanto-Ward E, Soler AP, Laury-Kleintop LD, Mandik-Nayak L, Metz R, Ostrand-Rosenberg S (2012). IDO is a nodal pathogenic driver of lung cancer and metastasis development. Cancer Discov.

[10] Prendergast GC (2008). Immune escape as a fundamental trait of cancer: focus on IDO. Oncogene.

[11] Luo J, Solimini NL, Elledge SJ (2009). Principles of cancer therapy: oncogene and non-oncogene addiction. Cell.

[12] Munn DH, Zhou M, Attwood JT, Bondarev I, Conway SJ, Marshall B, Brown C, Mellor AL (1998). Prevention of allogeneic fetal rejection by tryptophan catabolism. Science.

[13] Mei J, Li MQ, Ding D, Li DJ, Jin LP, Hu WG, Zhu XY (2013). Indoleamine 2,3-dioxygenase-1 (IDO1) enhances survival and invasiveness of endometrial stromal cells via the activation of JNK signaling pathway. Int J Clin Exp Pathol.

[14] Su C, Zhang P, Liu J, Cao Y (2017). Erianin inhibits indoleamine 2, 3-dioxygenase -induced tumor angiogenesis. Biomed Pharmacother.

[15] Macchiarulo A, Camaioni E, Nuti R, Pellicciari R (2009). Highlights at the gate of tryptophan catabolism: a review on the mechanisms of activation and regulation of indoleamine 2,3-dioxygenase (IDO), a novel target in cancer disease. Amino Acids.

[16] Karanikas V, Zamanakou M, Kerenidi T, Dahabreh J, Hevas A, Nakou M, Gourgoulianis KI, Germenis AE (2007). Indoleamine 2,3-dioxygenase (IDO) expression in lung cancer. Cancer Biol Ther.

[17] Yao Q, Cao Z, Tu C, Zhao Y, Liu H, Zhang S (2013). MicroRNA-146a acts as a metastasis suppressor in gastric cancer by targeting WASF2. Cancer Lett.

[18] Zhou X, Zhang L, Zheng B, Yan Y, Zhang Y, Xie H, Zhou L, Zheng S, Wang W (2016). MicroRNA-761 is upregulated in hepatocellular carcinoma and regulates tumorigenesis by targeting Mitofusin-2. Cancer Sci.

[19] Huang H, Hu M, Li P, Lu C, Li M (2015). Mir-152 inhibits cell proliferation and colony formation of CD133(+) liver cancer stem cells by targeting KIT. Tumour Biol.

[20] Yuan Y, Shen Y, Xue L, Fan H (2013). miR-140 suppresses tumor growth and metastasis of non-small cell lung cancer by targeting insulin-like growth factor 1 receptor. PLoS One.

[21] Shao J, Wang L, Zhong C, Qi R, Li Y (2016). AHSA1 regulates proliferation, apoptosis, migration, and invasion of osteosarcoma. Biomed Pharmacother.

[22] Ni QF, Tian Y, Kong LL, Lu YT, Ding WZ, Kong LB (2014). Latexin exhibits tumor suppressor potential in hepatocellular carcinoma. Oncol Rep.

[23] Shen Y, Tang D, Yao R, Wang M, Wang Y, Yao Y, Li X, Zhang H (2013). microRNA expression profiles associated with survival, disease progression, and response to gefitinib in completely resected non-small-cell lung cancer with EGFR mutation. Med Oncol.

